# Development and Evaluation of a Duo *Zaire ebolavirus* Real-Time RT-PCR Assay Targeting Two Regions within the Genome

**DOI:** 10.3390/microorganisms7120652

**Published:** 2019-12-04

**Authors:** Laurence Thirion, Remi N. Charrel, Yannik Boehmann, Iban Corcostegui, Hervé Raoul, Xavier de Lamballerie

**Affiliations:** 1 Unité des Virus Emergents (UVE: Aix Marseille Univ, IRD 190, INSERM U1207, IHU Méditerranée Infection), Aix Marseille Universite, Marseille 13000, France; laurence.thirion@ird.fr (L.T.); yannik.boehmann@gmx.com (Y.B.); iban.corcostegui@gmail.com (I.C.); xavier.de-lamballerie@univ-amu.fr (X.d.L.); 2 Laboratory P4-Jean Mérieux, INSERM, Lyon 69007, France; herve.raoul@inserm.fr

**Keywords:** filoviridae, ebola virus disease, diagnostics, emerging, viral hemorrhagic fever, preparedness, response.

## Abstract

Preparedness and response actions to mitigate Ebola virus disease (EVD) outbreaks rely on rapid diagnosis to be implemented locally to sort suspect patients attending health centers. Our aim was (i) to develop and evaluate an RT-qPCR assay combining primers and probes derived from two reference assays targeting different genomic regions; (ii) to study whether sensitivity and specificity of this dual-target assay were at least equal or better to the parental assays; (iii) to implement this dual-target assay onto the Cepheid GeneXpert open cartridge as a proof of principle for technological transfer aiming at bedsite testing locally. To do so, three home-made published RT-qPCR assays were selected to be compared with the RealStar^®^ Filovirus Screen RT-PCR kit 1.0 (Altona Diagnostics, Hamburg, Germany), a technique that was largely deployed during the 2014–2015 West African EVD outbreak. Primers and probes sequences of the custom-made assays were analyzed in silico against a multiple sequence alignment, including >250 complete sequences corresponding to strains that have caused EVD epidemics in the past. Genomic RNA purified from the Mekambo strain of *Zaire ebolavirus* (EBOV) was used to study the sensitivity of the five methods. Based on these results, two in-house methods were selected and adapted to design the dual-target assay, which performances were compared to those of the parental assays using a synthetic RNA control. The dual-target assay showed better sensitivity and limit of detection (LoD_95_ at 0.4 copies/µL) than the parental methods (1.7 and 2.2 copies/µL). Ultimately, the dual-target assay was transferred onto the GeneXpert Flex-03 open cartridge, demonstrating a LoD_95_ at 0.75 copies/µL. Together these results indicate that EBOV dual-target assay has the potential to be used during EVD outbreak in the laboratory having performed molecular testing during the recent outbreaks.

## 1. Introduction

The genus Ebolavirus (family Filoviridae) includes five species: *Zaire ebolavirus* (EBOV), *Sudan ebolavirus, Taï Forest ebolavirus, Reston ebolavirus* and *Bundibugyo ebolavirus* [1,2]. All are of African origin, except *Reston ebolavirus,* which originates from Asian [3]. EBOV has been responsible for the vast majority of human cases (i) in Central Africa (Democratic Republic of Congo, 1976-1977-1995-2002-2003-2007; Gabon, 1994-1996-1997-2001-2002; Republic of Congo, 2002–2003), and (ii) recently in Western Africa (Liberia, Sierra Leone, and Guinea, 2014–2016), the latter being by far the largest EBOV outbreak [4].

The recent Western African EBOV outbreak has emphasized the need for reliable diagnostic tools amenable directly in the field. This is of conspicuous medical and public health importance since false-negative results are likely to leave contagious patients without containment measures, and because false-positive results will result in placing non-infected patients in premises together with highly contagious Ebola virus disease (EVD) patients. Hence, molecular diagnosis has demonstrated their capacity to be deployed and implemented in mobile laboratories where their high sensitivity and specificity combined with the potential to provide results within few hours are pivotal for sorting suspect patients attending the health centers. They remain, to date, the first-intention diagnostic techniques, but they still require development to be used by routine technical staff locally and merit to be not only sensitive and specific but prepared for easy read-out and for the possible occurrence of mutant strains during an on-going outbreak [5].

The purposes of the present study was (i) to compare three reference home-made RT-qPCR assays for the detection of EBOV with commercial reference assays; (ii) to develop a dual-target RT-qPCR assay combining two of the aforementioned single-target assays as recently described for Chikungunya virus [6], and (iii) to attempt transferring this dual-target RT-qPCR assay into a Cepheid GeneXpert open cartridge.

## 2. Materials and Methods

### 2.1. In Silico Analysis

Primers and probes sequences of the custom-made assays were analyzed in silico against a multiple sequence alignment, including >235 complete sequences corresponding to strains having caused EVD epidemics from the first DRC (Democratic Republic of Congo) strain in 1976 to the strains having caused the West African outbreak from 2014 to 2015. Alignments were performed with MEGA 6.0 and MUSCLE softwares [7,8]. For identical sequences, only the first sequence was retained for further analysis. Sequences of primers and probes of the selected assays were also aligned against the multiple sequence alignment to study the presence of mismatches that may hamper the detection of certain strains. Using the same approach, primers and probes were matched against the most recent strains of EBOV listed in the supplementary material (Figures S1 and S2).

### 2.2. Mekambo EBOV RNA

Genomic RNA from the Mekambo strain of EBOV (Medemba village, Gabon, 2001 [1,2]) was prepared at Laboratory P4-Jean Mérieux, INSERM, Lyon, France. The virus was grown in Vero cells and viral RNA was extracted from 100 µL of cell culture supernatant and purified using the QIAcube HT Robot using the Cador Pathogen 96 kit (Qiagen, Courtaboeuf, France) as recommended. Elution was performed in 80 µL of AVE buffer (Qiagen, Courtaboeuf, France), aliquoted and stored at −80 °C. Mekambo EBOV RNA concentration (3.4 × 10^5^ copies per µL) was quantified by RT-qPCR performed with GoTaq Probe 1-Step RT-qPCR (Promega, Charbonnières-les-Bains, France).

### 2.3. EBOV RNA Transcript for LoD Calculation

A custom-made synthetic RNA control was designed to include genome regions corresponding to the assays developed for the three assays included in the study (Gibb et al., Huang et al., and Panning et al.) [9,10,11] The transcript was produced by using the MEGAshortscript™ T7 Transcription Kit (Ambion™) according to the manufacturer’s protocol. Plasmid DNA was removed with the DNase, and the RNA transcript was purified by using Monarch^®^ PCR & DNA Cleanup Kit (New England Biolabs, Evry, France)). The RNA concentration was measured by using a Thermo Scientific™ NanoDrop™, and the copy number, 5 × 10^12^ copies/µL, was calculated by using the molecular weight of the RNA. The RNA transcript was serially diluted 100-fold, and each diluted standard was stored at −80 °C until used.

### 2.4. RT-qPCR Assays

Home-made RT-qPCR assays are described in Table 1. RT-qPCR assays were performed on Bio-Rad CFX96™ cyclers. All assays were done by using the standard protocol (Table 2) with GoTaq Probe 1-Step RT-qPCR (Promega), in a 25 µL total reaction volume including 5 µL Mekambo EBOV RNA or 30 µL total reaction volume including 10 µL of EBOV synthetic RNA. The three home-made RT-qPCR assays were compared with the RealStar^®^ Filovirus Screen RT-PCR kit 1.0 (Altona Diagnostics, Hamburg, Germany). Tests using Mekambo EBOV RNA were performed with a ten-fold serial dilution ranging from 10^5^ to 1 copy per microliter. Tests using the synthetic RNA standard were performed with 5-fold serial dilution ranging from 1 to 0.02 copy per microliter. Seven replicates were tested for each dilution with the three home-made single-target assays and 23 replicates per dilution for the Duo Gibb + Huang RT-qPCR (Gibb/Huang) assay. EBOV synthetic RNA was used to determine the limit of detection (LoD). The LoD is defined as the lowest concentration or amount of analyte required to produce a positive assay in at least 95% of the replicates. LoD was calculated using the IBM SPSS statistic 21 software.

### 2.5. Specificity

Strains of filoviruses (*n* = 2), flaviviruses (*n* = 9), alphaviruses (*n* = 7), phleboviruses (*n* = 2), and nairoviruses (*n* = 1) were selected (Table 3). All the viral strains included in the specificity panel were provided by European Virus Archive Goes Global (EVAg, Available online: https://www.european-virus-archive.com/), except Marburg and Crimean-Congo hemorrhagic fever virus RNA kindly provided by the BSL4 INSERM-Jean Merieux laboratory in thiocyanate guanidinium.

### 2.6. Cepheid GeneXpert Open Cartridge Development

We tested the duplex multigenic assay combining the Gibb and Huang assays onto the GeneXpert RT-qPCR open cartridge. Tests were performed with the EBOV synthetic RNA using serial dilutions ranging from 16.8 to 1.7 copies/reaction. Eight replicates were done for each dilution. All RT-qPCR assays were carried onto a GeneXpert^®^ GX-IV (Cepheid, USA). Each reaction contained a total volume of 85 µL (12.47 μL of primers and probes, 39.73 μL of RE buffer, 28.5 μL of RNA to which was added 4.47 μL of MgSO_4_, and a lyophilized enzyme bead provided by Cepheid. The 85 µL were introduced into chamber #11 of the open cartridge. The qRT-PCR assay consisted of a 15-min reverse transcription at 42 °C, a 2-min activation at 95 °C, then 45 cycles consisting of 96 °C for 5 s and 60 °C for 35 s.

## 3. Results

### 3.1. RT-qPCR Primers and Probe Matched against EBOV Multiple Sequence Alignment: In Silico Analysis

**Figure 1:**
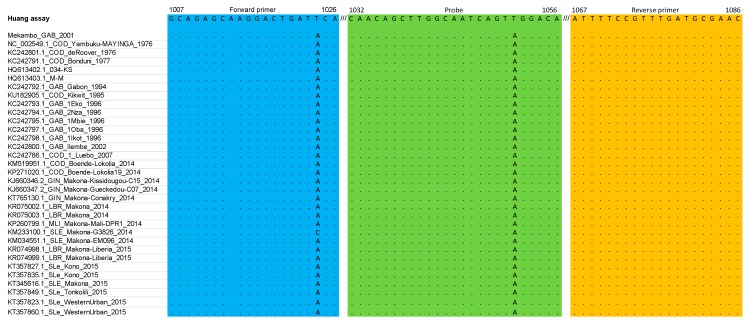
Primers and probe described in the Huang et al. [10] assay matched against the multiple alignment dataset (nucleoprotein gene).

**Figure 2:**
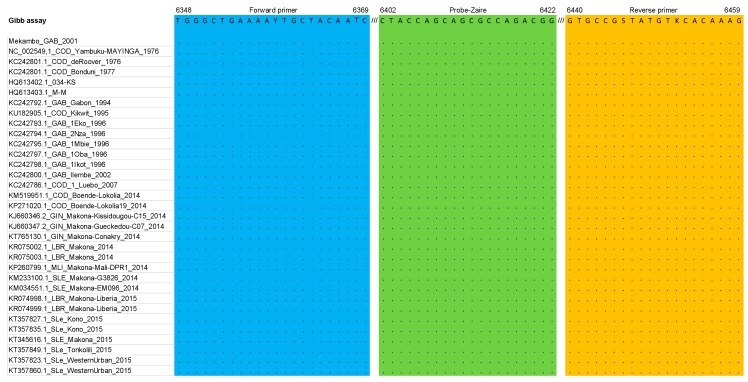
Primers and probe described in the Gibb et al. [9] assay matched against the multiple alignment dataset (glycoprotein gene); Y = C or T, S = C or G, K = G or T.

**Figure 3:**
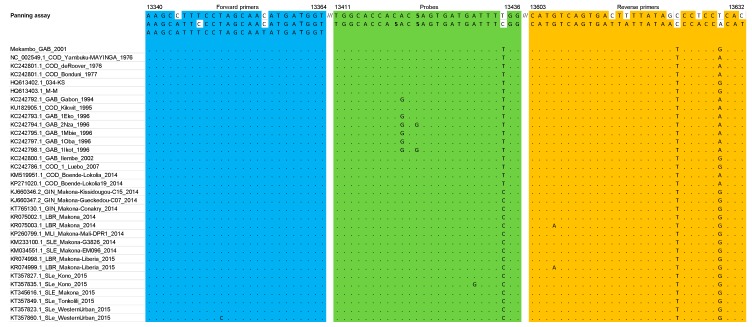
Primers and probe described in the Panning et al. [11] assay matched against the multiple alignment dataset (*RdRp* gene).

**Figure 4:**
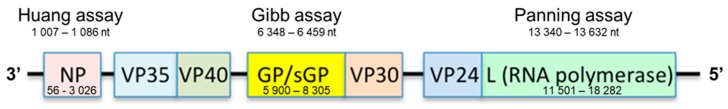
Schematic representation of the *Zaire ebolavirus* (EBOV) genome with the regions targeted by the three assays.

Figure 1, Figure 2 and Figure 3 summarize the stringency of the primers and probes described in the three selected home-made assays. Figure 4 presents a schematic representation of the EBOV genome with the targeted regions of the three assays.

In the Huang et al. [10] assay, the occurrence of a mismatch close to the 3′ end of the sense primer (enp-F) that was not covered by a wobble in the primer sequence, led to the design of a second primer (enp-F2, GCAGAGCAAGGACTGATACA) to avoid mismatches with possible deleterious effects on the performances of the assay. A single mismatch was observed within the probe, but it was decided not to modify the original sequence because it was positioned on the 6th nucleotide from the 3′-end. No mismatch was observed in the reverse primer.

In the Gibb et al. assay [9], no mismatch was observed either in the sense and reverse primers or in the probe.

In the Panning et al. [11] assay, the FiloA2.4 forward primer showed a 100% identity with ZEBOV and the Mekambo sequence. The combination of the two probes allowed the genetic diversity to be covered; hence, they were not modified. Due to mismatches observed at positions 21 and 27 in the sequence of the Filo B reverse primer, we designed a Filo B-prime reverse primer (CATGTCAGTGATTATTATAAYCCACCRCAT) to be included in the final mix, which incorporated Y and R wobbles at position 21 and 27, respectively.

### 3.2. Study Using the Mekambo EBOV RNA

Intra- and inter-assays variability was determined using six dilutions (10^5^ to 1 copy of Mekambo EBOV RNA per µL) and seven replicates for each of the three home-made RT-qPCR assays and for the Duo Gibb + Huang RT-qPCR, or four replicates for the Altona RealStar assay (Figure 4). The reproducibility was excellent for the five assays with slight Ct variations. The standard deviations ranged from 0.4 to 0.8 with the Gibb et al. [9] assay, 0.1 to 1.3 with the Huang et al. [10] assay, and 0.2 to 1.7 with the Panning et al. [11] assay, 0.2 to 1.5 with the Duo Gibb + Huang assay, and 0.3 to 1.5 with the Altona RealStar assay.

**Figure 5:**
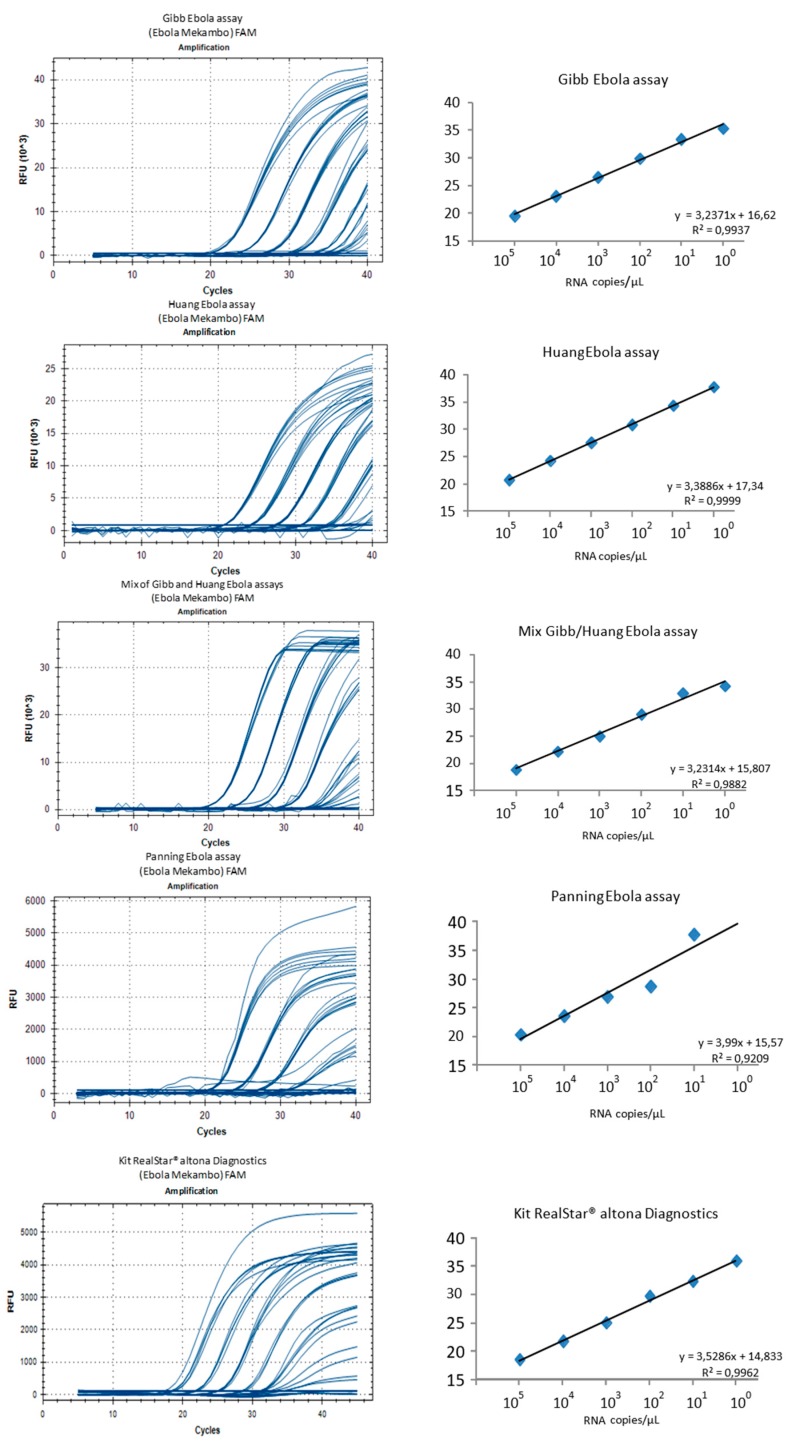
Comparison of five assays using serial dilutions of EBOV Mekambo RNA ranging from 10^5^ to 1 copy per microliter.

The limit of detection for 1 Ebola Mekambo genome copy per reaction was observed for all the tested assays except Panning et al. [11]. For the latter, the results observed with concentrations ranging from 10^5^ to 10^3^ genome copies per microliter are similar to the other assays, but results are less satisfactory with 100 and 10 copies per microliter and missed the 1 copy per microliter. All other assays provided equivalent results suggesting that the Duo Gibb + Huang assay is an alternative to be considered compared with assays targeting a single region of the genome. The relative fluorescence unit (RFU) varied significantly between the tested assays with the highest values (>20,000 at 10^5^ and >10,000 at 10) observed with the Gibb et al. [9], Huang et al. [10], and Duo Gibb + Huang assays, whereas much lower values (<5000 at 10^5^ and <3000 at 10) were observed with Panning et al. [11] and Altona RealStar assay (Figure 5).

### 3.3. LoD Calculation Using the EBOV Synthetic RNA

**Figure 6:**
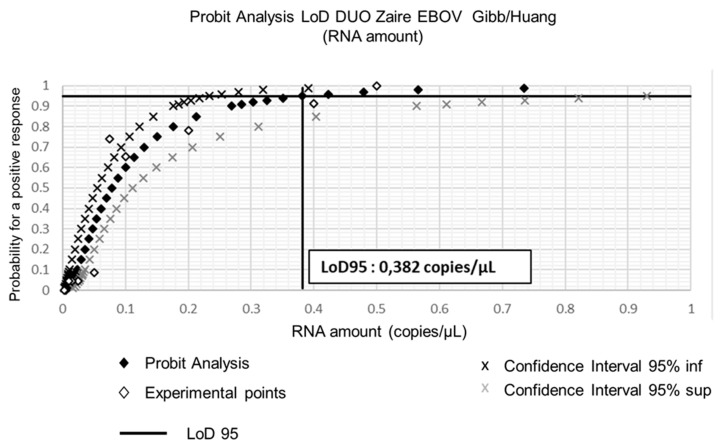
Limit of detection of the Duo Gibb + Huang assay using EBOV synthetic RNA.

**Figure 7:**
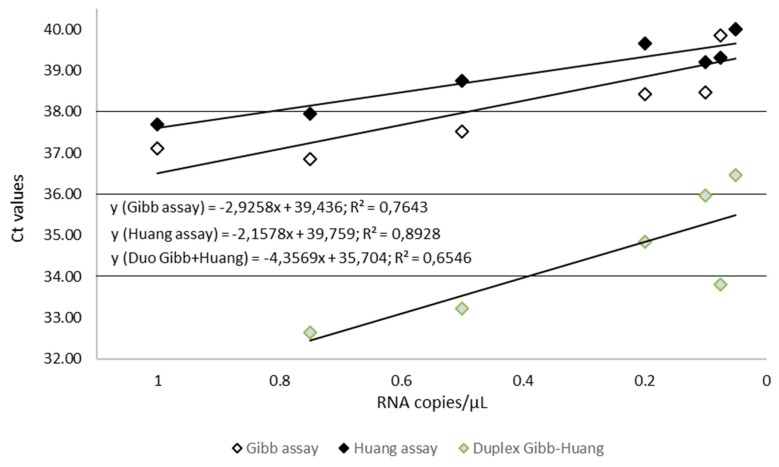
Comparison of standard curves for Gibb, Huang, and Gibb/Huang assays using serial diluted EBOV RNA transcript, dilution range from 1 to 0.02 copy per microliter. The RNA copy numbers per reaction are plotted on the X-axis, and the cycle number crossing point values (Ct values) are plotted on the Y-axis. Ct, Cut-off threshold.

To assess more precisely the sensitivity of the in-house assays that provided the best results during the first stages of this study, we used dilutions of the quantified EBOV synthetic RNA. The aim was to estimate the limit of detection (LoD) for both Gibb et al. [9] and Huang et al. [10] assays and to calculate the LoD of the Duo Gibb + Huang assay. Results presented in Table 3 and Figure 5 show that the LoD_95_ of the Duo Gibb + Huang assay was lower (0.382 RNA copies/µL)) compared with those provided by Gibb et al. [9] (1.7 RNA copies/µL) and Huang et al. [10] assays (2.2 RNA copies/µL) (Figure 6). In contrast, the linearity of the Duo Gibb + Huang assay was lower than that of the two other assays (Figure 7).

### 3.4. Specificity of the Duo Gibb + Huang Assay

The specificity of the assay was tested against 21 strains of several related and non-related viruses from *Filovirus*, *Alphavirus*, *Flavivirus*, *Nairovirus,* and *Phlebovirus* genera. None of these 21 target RNA was amplified using the Duo Gibb + Huang assay.

### 3.5. Transfer of the Duo Gibb + Huang Assay onto the Flex-03 Cartridge and Validation on the GeneXpert (Cepheid)

All replicates performed with the Flex-03 open cartridge on the GeneXpert were positive at 16.8 and 12.6 RNA copies/reaction (Table 4) corresponding respectively to 1 and 0.75 copies/µL (Table 5). The LoD_95_ was less than 0.75 copies/µL on eight independent runs. When compared with the LoD_95_ obtained using the Duo Gibb + Huang as aforementioned, the results are in the same order of magnitude (0.75 vs. 0.382).

Assays repeatability was assessed by calculating the standard deviation (SD) for the Ct variance, presented in Table 5. Globally, observed SD are low, which is suggest that repeatability is acceptable, although this merits assessment by specific experiments with a larger number of replicates.

## 4. Discussion

Although EBOV has been first isolated and identified more than 50 years ago, sorting suspect patients by using real-time RT-PCR-based diagnostics remains challenging in the region where outbreak are on-going. There are a growing number of molecular tests described in the literature. The tests that were selected in this study have been widely used not only in epidemic situations but also in external quality assessment studies [9,10,11,12,13,14,15,16,17,18,19,20]. Dual-target assays are increasingly used in commercial and in-house assays [6,21,22,23]. Dual-target assays, also known as Duo assays (i) allow the detection of viral variants resulting in genomes with mutated regions which might affect hybridization of either primers or probe of the original assay; (ii) simplify the interpretation of the results due to the use of a unique fluorescent dye for different probes included in the assay, and (iii) frequently improves the sensitivity as previously shown with Chikungunya virus [6]. Here, we selected four assays that were targeting different regions of the genome (*NP* and *GP* genes) and which provided the most interesting in silico results.

### 4.1. In Silico Analysis

Of the three assays that were selected to be investigated, the Gibb et al. [9] and Huang et al. [10] assays were chosen to develop the Duo RT-qPCR assay. Because of the identification of a mismatch at the 3′ end of the sense primer in the Huang et al. [10] assay, an additional primer was designed and included in the subsequent testing, not only in the Duo assay but also in the single-plex assay for comparative analysis. The Gibb et al. [9] assay was used unchanged (Figure 1, Figure 2 and Figure 3). From 2015 to now, EBOV has caused new epidemics in DRC and Uganda, totaling at least 3606 cases with 2156 deaths; sequences of these recent strains were also theoretically almost perfectly detected based on the in silico analysis (Figures S1 and S2).

### 4.2. Sensitivity of the Duo Gibb + Huang Assay

All but the Panning et al. [11] assay, demonstrated their capability to detect 1 RNA copy/µL; this is the reason why the Panning et al. [11] assay was not retained for further experiments. To investigate further the sensitivity and limit of detection of these assays, a synthetic RNA control was designed. Since sequence data were not publicly available for the Altona RealStar assay, the latter was also not retained for subsequent studies, which were ultimately centered on the Duo Gibb + Huang assay and on the two parental assays.

The Duo Gibb + Huang assay showed a sensitivity that was at least equal, and even better than that observed with the two parental assays. The LoD was better for the Duo Gibb + Huang assay than LoDs observed for both parental assays, independently. In addition, the Duo assay demonstrated higher RFU, as previously described with CHIKV Duo [6], that warrants an easier interpretation of the results for low-copy samples, which are notoriously the most difficult situation at the validation stage. This is of particular importance because molecular detection of EBOV RNA is the cornerstone for sorting patients suspect of EVD. The reason for increased RFU observed with Duo assays is not fully elucidated; our hypothesis is it could be linked to the superposition of two labeled probes accounting for a higher signal.

### 4.3. Transfer of the Duo Gibb + Huang Assay onto the Flex-03 Cartridge and Validation on the GeneXpert (Cepheid)

Interestingly, Cepheid has developed a closed cartridge for the detection of the Ebola virus (Xpert^®^ Ebola) by targeting the *NP* and the *GP* genes from whole blood. The rationale for using two target genes was to lower the risk of false-positive results for new variants. Depending on the material used for evaluation (EBOV RNA, inactivated EBOV, or infectious EBOV), the LoD ranged from 73 to 232 copies/mL (95% CI: 51–302 copies/mL). The assay correctly identified five different EBOV strains, Yambuku-Mayinga, Makona-C07, Yambuku-Ecran, Gabon-Ilembe, and Kikwit-956210, and correctly excluded all non-EBOV isolates tested [24]. Subsequently, the Xpert^®^ Ebola assay was used in the comparative study versus TaqMan RT-qPCR assays [25]) and in field studies [26,27,28]. Our aim in this study was not to challenge the Xpert^®^ Ebola with the Duo Gibb + Huang assay but to evaluate how complex it would be to transfer a test that was operational in a classic real-time TaqMan platform onto a Flex open cartridge provided with the Noro Bead EZR enzyme beads. The advantage of the GeneXpert system is that it integrates all the steps required to perform sample-processing and real-time PCR, including internal control, into a single plastic cartridge. The cartridge is used in a single automated test unit. The LoD 95 was less than 0.75 copies/µL, the limit of detection was comparable to that obtained, 0.4 copies/µL, with a “classic” thermocycler. We are perfectly aware that additional and more detailed testing is necessary before the Flex-cartridge might be used for the detection of EBOV in the field; however, the first results are promising and support further development and evaluation. The combination of the Duo Gibb + Huang primers and probes together with the Flex cartridge and the Noro Bead EZR should be considered as an important piece in the arsenal of diagnostics prepared for mitigating epidemic situations caused by re-emerging pathogens, in particular, those for which the diagnostic capacities are scarce and for which field usage is important in the quality of the response plan. Although our study has clear limitations, this is, to the best of our knowledge, the first time that such a transfer has been explored.

## 5. Conclusions

Here, we combined two assays that were targeting different regions of the genome (*NP* and *GP* genes) and which provided the most interesting in silico results. This study demonstrates that developing a Duo assay from two previously validated assays is easy and rapid. Within this context, the resulting Duo assay proved more sensitive than the two parental assay, independently. We also showed that this Duo assay is amenable to be used onto the GeneXpert Flex cartridge, on which very promising results were obtained as a proof of principle that must be considered seriously in emergency situations when molecular diagnosis must be brought at the patient bedside.

## Figures and Tables

**Table 1:** Oligonucleotide primers and probes used in this study. Supplementary primers designed in this study are bolded.

Assay	Target	Amplicon	Primers/Probe	Sequence (5′-3′)
Gibb	*GP*	112 bp	EBOGP1D-fwd	TGGGCTGAAAAYTGCTACAATC
			EBOGP1D-rev	GTGCCGSTATGTKCACAAAG
			EBOGP1DZ-Prb	FAM-CTACCAGCAGCGCCAGACGG-TAMRA
Huang	*NP*	80 bp	enp-F	GCAGAGCAAGGACTGATTCA
			**Enp-F2**	**GCAGAGCAAGGACTGATACA**
			enp-R	ATTTTCCGTTTGATGCGAAC
			enp-Probe	FAM^a^-CAACAGCTTGGCAATCAGTTGGACA-TAMRA
Panning	*L*	293 bp	FiloA2.2	AAGCCTTTCCTAGCAACATGATGGT
			FiloA2.3	AAGCATTCCCTAGCAACATGATGGT
			FiloA2.4	AAGCATTTCCTAGCAATATGATGGT
			FiloB	CATGTCAGTGATTATTATAACCCACCACAT
			**Filo B-Prime**	**CATGTCAGTGATTATTATAAYCCACCRCAT**
			Filo B-Ra	CATGTCAGTGACTTTTATAGCCCTCCTCAC
			FAMEBOSu	FAM^b^-TGGCACCAIACIAGTGATGATTTCGG-BHQ1
			FAMEBOg	FAM^b^-TGGCACCACACIAGTGATGATTTTGG-BHQ1

FAM, 6-carboxyfluorescein reporter dye. Bolded, designed in this study.

**Table 2:** Conditions of the reactions as described in the original studies and using the standard protocol.

	Gibb et al. [9]	Huang et al. [10]	Panning et al. [11]	Standard Protocol Mekambo EBOV RNA	Standard Protocol EBOV Synthetic RNA	RealStar^®^ Filovirus Altona Diagnostics
Reverse Transcription	55 °C/45 min	50 °C/30 min	50 °C/30 min	50 °C/15 min	50 °C/15 min	55 °C/20 min
Denaturation	94 °C/1 min	94 °C/5 min	95 °C/1.5 min	95 °C/2 min	95 °C/2 min	95 °C/2 min
Cycling	40	45	45	40	40	45
Denaturation	94 °C/15 s	94 °C/15 s	95 °C/15 s	95 °C/15 s	95 °C/15 s	95 °C/15 s
Amplification	60 °C/30 s	60 °C/1 min	72 °C/20 s	60 °C/1 min	60 °C/1 min	72 °C/15 s
RNA volume	5 µL	1–3 µL	3 µL	5 µL	10 µL	10 µL
Total volume	50 µL	23–25 µL	25 µL	25 µL	30 µL	30 µL
Primers concentration	0.5 µM	0.4 µM	0.2 µM Forward 0.3 µM Reverse	0.4 µM Gibb 0.4 µM Huang 0.2 µM Panning	0.625 µM Gibb 0.5 µM Huang	-
Probes concentration	0.2 µM	0.1 µM	0.0667 µM	0.16 µM Gibb and Huang	0.25 µM Gibb	-
				0.2 µM each Panning	0.125 µM Huang	

**Table 3:** Viral strains tested to evaluate the specificity of the Duo Gibb + Huang *Zaire ebolavirus* (EBOV) RT-qPCR.

Genus	Virus	Acronyms	Strain (Viral Load TCID50/mL)	Reference (a)
*Phlebovirus*	Toscana virus	TOSV	UVE/TOSV/2014/FR/5904 (10 ^8,22^)	001v-02452
	Sandfly Fever Sicilian virus	SFSV	UVE/SFSV/1943/IT/Sabin (10 ^6,82^)	001v-EVA77
*Flavivirus*	Japanese encephalitis virus	JEV	UVE/JEV/2009/LA/CNS769 (10 ^5,57^)	001v-02217
	Saint-Louis encephalitis virus	SLEV	UVE/SLEV/UNK/US/MSI-7 (10 ^4,82^)	001v-EVA128
	Tick-borne encephalitis virus	TBEV	UVE/TBEV/2013/FR/32.11 WT-PCR (10 ^8,82^)	001v-02352
	West-Nile virus	WNV	UVE/WNV/2008/US/R94224 (10 ^7,32^)	001v-02224
	Yellow Fever virus	YFV	UVE/YFV/UNK/XX/French neurotropic R94224 (10 ^7,32^)	001v-02226
	Usutu virus	USUV	UVE/USUV/1959/ZA/SAAR-1776 (10 ^5,32^)	001v-EVA138
	Murray Valley virus	MVEV	UVE/MVEV/UNK/AU/3329 (10 ^4,32^)	001v-EVA145
	Zika virus	ZIKV	UVE/ZIKV/1947/UG/MR766 (10 ^4,32^)	001v-EVA143
	Dengue virus	DENV-1	UVE/DENV-1/2013/NC/CNR_17132 (10 ^7,57^)	001v-03151
*Alphavirus*	Venezuelan equine encephalitis virus	VEEV	UVE/VEEV/UNK/XX/TC83 vaccine (10 ^9,42^)	001v-EVA1459
	Western equine encephalitis virus	WEEV	UVE/WEEV/UNK/XX/47a (10 ^8,32^)	001v-EVA1479
	Eastern equine encephalitis virus	EEEV	UVE/EEEV/1999/XX/H178_99 (10 ^7,82^)	001v-EVA1480
	O’nyong-nyong virus	ONNV	UVE/ONNV/UNK/SN/Dakar 234 (10 ^4,22^)	001v-EVA1044
	Chikungunya virus	CHIKV	UVE/CHIKV/2017/FR/45625-26 (10 ^6,16^)	001v-03433
	Semliki Forest virus	SFV	UVE/SFV/UNK/XX/1745 (10 ^4,42^)	001v-02468
	Sindbis virus	SINV	UVE/SINV/UNK/EG/Egypt 339 (10 ^4,32^)	001v-02469
*Filovirus*	Marburg virus	MBGV	Popp	n/a
	Marburg virus	MBGV	Musoke	n/a
*Nairovirus*	Crimean-Congo hemorrhagic fever virus	CCHF	Unk	n/a

(a), reference on the European Virus Archive website (Available online: https://www.european-virus-archive.com/); n/a, not applicable.

**Table 4:** Ct values at different synthetic RNA concentrations.

Replicate	RNA Copies/Reaction				
	16.8	12.6	8.4	4.2	1.7
Flex-C#1	37.1	38.7	39.6	negative	negative
Flex-C#2	36.3	36.0	38.9	38.0	negative
Flex-C#3	36.4	36.9	39.2	negative	negative
Flex-C#4	38.4	37.6	37.9	negative	negative
Flex-C#5	36.2	38.2	negative	36.7	negative
Flex-C#6	36.5	39.3	negative	38.0	negative
Flex-C#7	39.7	37.8	negative	negative	negative
Flex-C#8	36.1	36.2	negative	negative	negative
Mean	37.1 (1.3)	37.6 (1.2)	38.9 (0.6)	37.6 (0.6)	-

**Table 5:** Comparison of the limit of detection (LoD) observed with serial dilutions of the synthetic EBOV RNA control using the Duo Gibb + Huang assay compared with the two original single-plex assays. Values in the Table are Ct values, Ct values >40 are considered as negative; Ct, Cut-off threshold.

**GIBB et al.** [9]	**Replicate**	**RNA Copies/µL**						
		1	0.75	0.5	0.2	0.1	0.075	0.05
	1	36.57	36.66	37.28	38.76	>40	>40	>40
	2	36.59	35.99	37.25	38.43	>40	>40	>40
	3	37.02	36.62	36.98	>40	38.36	>40	>40
	4	36.47	37.07	>40	38.27	>40	>40	>40
	5	38.47	36.17	>40	38.27	>40	>40	>40
	6	37.49	>40	38.54	>40	38.54	>40	>40
	7	>40	38.53	37.59	>40	38.52	39.85	>40
	Mean Ct (SD)	37.10 (0.7)	36.84 (0.8)	37.53 (0.5)	38.43 (0.2)	38.47 (0.1)	39.85	>40
**HUANG et al.** [10]	**Replicate**	**RNA Copies/µL**						
		1	0.75	0.5	0.2	0.1	0.075	0.05
	1	37.59	>40	38.14	>40	38.60	>40	>40
	2	37.92	38.02	>40	40	>40	>40	>40
	3	37.24	38.42	39.62	>40	39.09	>40	>40
	4	37.30	38.48	>40	>40	>40	37.95	>40
	5	38.35	37.39	>40	>40	>40	39.98	>40
	6	37.89	>40	38.07	>40	>40	39.98	>40
	7	37.48	37.48	39.13	39.31	39.92	>40	>40
	Mean Ct (SD)	37.68 (0.4)	37.96 (0.5)	38.74 (0.7)	39.66	39.20 (0.5)	39.30 (1.0)	>40
**DUO GIBB + HUANG (this study)**	**Replicate**	**RNA Copies/µL**						
		1	0.75	0.5	0.2	0.1	0.075	0.05
	1	NT	32.14	33.04	34.89	36.40	32.98	>40
	2	NT	33.01	32.78	34.93	36.24	33.17	>40
	3	NT	32.87	33.10	34.03	>40	33.80	38.27
	4	NT	32.83	33.20	34.88	>40	33.48	>40
	5	NT	32.77	36.61	34.67	37.45	> 40	>40
	6	NT	32.28	33.53	35.63	35.34	32.95	>40
	7	NT	32.71	32.74	>40	35.43	>40	>40
	8	NT	32.59	32.84	34.64	35.18	33.41	>40
	9	NT	31.98	33.40	34.04	35.39	33.97	>40
	10	NT	32.44	33.12	35.12	>40	33.01	>40
	11	NT	33.11	33.33	34.67	35.88	33.83	>40
	12	NT	32.27	32.73	35.36	35.36	33.56	>40
	13	NT	32.19	33.45	>40	38.37	36.71	>40
	14	NT	32.41	33.04	34.29	>40	34.74	>40
	15	NT	33.06	33.29	34.48	36.04	34.18	>40
	16	NT	32.47	32.41	35.69	>40	>40	34.65
	17	NT	32.07	32.93	34.70	>40	33.18	>40
	18	NT	32.22	33.15	>40	35.86	32.29	>40
	19	NT	34.00	33.46	36.39	35.78	36.10	>40
	20	NT	32.38	34.32	>40	33.92	33.17	>40
	21	NT	33.12	33.05	34.57	36.72	>40	> 40
	22	NT	32.44	32.55	34.31	>40	>40	>40
	23	NT	33.08	32.23	>40	> 40	>40	>40
	Mean Ct (SD)		32.63 (0.5)	33.23 (0.8)	34.85 (0.6)	35.96 (1.0)	33.80 (1.1)	36.46 (1.8)

## Supplementary Materials

Supplementary materials can be found at https://www.mdpi.com/2076-2607/7/12/652/s1, Figure S1: Primers and probe described in the Huang et al. assay (and supplementary forward primer [this study]) matched against the multiple alignment dataset (nucleoprotein gene) representing EBOV strains during the 2018 outbreak in the North Kivu and Ituri Provinces of the Democratic Republic of the Congo. Y = C/T; S = C/G; K = G/T. Nucleotide positions refers to the sequence of Zaire ebolavirus genome (GenBank accession number MK007339). Figure S2: Primers and probe described in the Gibb et al. assay matched against the multiple alignment dataset (glycoprotein gene) representing EBOV strains during the 2018 outbreak in the North Kivu and Ituri Provinces of the Democratic Republic of the Congo. Y = C/T; S = C/G; K = G/T. Nucleotide positions refers to the sequence of Zaire ebolavirus genome (GenBank accession number MK007339).
