# Supplementary material for: Development and Evaluation of a Duo Zaire ebolavirus Real-Time RT-PCR Assay Targeting Two Regions within the Genome
Source: Microorganisms. 2019 Dec 4;7(12):652. doi: 10.3390/microorganisms7120652 (PMC6956279; doi:10.3390/microorganisms7120652)
Supplement: Supplementary file 1 [file microorganisms-07-00652-s001.pdf]

Supplementary

Huang assay (modified)			
	1007 Forward primer 1026	1032 Probe 1056	1067 Reverse primer 1086
	GCAGAGCAAGGACTGATTCA ///	CAACAGCTTGGCAATCAGTTGGACA ///	ATTTTCCGTTTGATGCGAAC
	GCAGAGCAAGGACTGATACA		
MK163662.1_COD_2018_Ituri-BEN225	.................A..	...................A.....	.....T..............
MK163647.1_COD_2018_Ituri-BEN031	.................A..	...................A.....	.....T..............
MK007337.1_COD_2018_Ituri-MAN036	.................A..	...................A.....	.....T..............
MK007331.1_COD_2018_Ituri-MAN002	.................A..	...................A.....	.....T..............
MK007338.1_COD_2018_Ituri-MAN037	.................A..	...................A.....	.....T..............
MK007334.1_COD_2018_Ituri-MAN020	.................A..	...................A.....	.....T..............
MK007333.1_COD_2018_Ituri-MAN006	.................A..	...................A.....	.....T..............
MK163644.1_COD_2018_Ituri-BEN017	.................A..	...................A.....	.....T..............
MK163649.1_COD_2018_Ituri-BEN104	.................A..	...................A.....	.....T..............
MK163650.1_COD_2018_Ituri-BEN114	.................A..	...................A.....	.....T..............
MK163656.1_COD_2018_Ituri-BEN162	.................A..	...................A.....	.....T..............
MK007330.1_COD_2018_Ituri-18FHV090	.................A..	...................A.....	.....T..............
MK007341.1_COD_2018_Ituri-MAN042	.................A..	...................A.....	.....T..............
MK731990.1_COD_2018_BEN164-MANA1-17	.................A..	...................A.....	.....T..............
MK007342.1_COD_2018_Ituri-MAN046	.................A..	...................A.....	.....T..............
MK007343.1_COD_2018_Ituri-MAN048	.................A..	...................A.....	.....T..............
MK007332.1_COD_2018_Ituri-MAN003	.................A..	...................A.....	.....T..............
MK007344.1_COD_2018_Ituri-MAN065	.................A..	...................A.....	.....T..............
MK007340.1_COD_2018_Ituri-MAN040	.................A..	...................A.....	.....T..............
MK007339.1_COD_2018_Ituri-MAN039	.................A..	...................A.....	.....T..............
MK007335.1_COD_2018_Ituri-MAN025	.................A..	...................A.....	.....T..............
MK088510.1_COD_2018_Ituri-MAN035	.................A..	...................A.....	.....T..............
MK163645.1_COD_2018_Ituri-BEN018	.................A..	...................A.....	.....T..............
MK163663.1_COD_2018_Ituri-BEN230	.................A..	...................A.....	.....T..............
MK163646.1_COD_2018_Ituri-BEN020	.................A..	...................A.....	.....T..............
MK163652.1 _COD_2018_Ituri-BEN141	.................A..	...................A.....	.....T..............
MK731993.1_COD_2018_BEN167-MANA5-11	.................A..	...................A.....	.....T..............
MK163653.1_COD_2018_Ituri-BEN143	.................A..	...................A.....	.....T..............
MK163654.1_COD_2018_Ituri-BEN146	.................A..	...................A.....	.....T..............
MK731992.1_COD_2018_BEN165-MANA3-20	.................A..	...................A.....	.....T..............
MK163648.1_COD_2018_Ituri-BEN070	.................A..	...................A.....	.....T..............
MK163651.1_COD_2018_Ituri-BEN140	.................A..	...................A.....	.....T..............
MK007329.1_COD_2018_Ituri-18FHV089	.................A..	...................A.....	.....T..............
MK163655.1_COD_2018_Ituri-BEN148	.................A..	...................A.....	.....T..............
MK163664.1_COD_2018_Ituri-BEN234	.................A..	...................A.....	.....T..............
MK163666.1_COD_2018_Ituri-BEN240	.................A..	...................A.....	.....T..............
MK163671.1_COD_2018_Ituri-BEN269	.................A..	...................A.....	.....T..............
MK163670.1_COD_2018_Ituri-BEN268	.................A..	...................A.....	.....T..............
MK163673.1_COD_2018_Ituri-BEN286	.................A..	...................A.....	.....T..............
MK163675.1_COD_2018_Ituri-BEN294	.................A..	...................A.....	.....T..............
MK163674.1_COD_2018_Ituri-BEN292	.................A..	...................A.....	.....T..............
MK163672.1_COD_2018_Ituri-BEN274	.................A..	...................A.....	.....T..............
MK163665.1_COD_2018_Ituri-BEN236	.................A..	...................A.....	.....T..............
MK163669.1_COD_2018_Ituri-BEN267	.................A..	...................A.....	.....T..............
MK163667.1_COD_2018_Ituri-BEN255	.................A..	...................A.....	.....T..............
MK163661.1_COD_2018_Ituri-BEN214	.................A..	...................A.....	.....T..............
MK163668.1_COD_2018_Ituri-BEN263	.................A..	...................A.....	.....T..............

Figure S1. Primers and probe described in the Huang et al. assay (and supplementary forward primer [this study]) matched against the multiple alignment dataset (nucleoprotein gene) representing EBOV strains during the 2018 outbreak in the North Kivu and Ituri Provinces of the Democratic Republic of the Congo. Y = C/T; S = C/G; K = G/T. Nucleotide positions refers to the sequence of Zaire ebolavirus genome (GenBank accession number MK007339).

Gibb assay					
	6348 Forward primer 6369		6402 Probe 6422		6440 Reverse primer 6459
	TGGGCTGAAAAYTGCTACAATC	///	CTACCAGCAGCGCCAGACGG	///	GTGCCGSTATGTKCACAAAG
MK163662.1_COD_2018_Ituri-BEN225	....		....		....
MK163647.1_COD_2018_Ituri-BEN031	....		....		....
MK007337.1_COD_2018_Ituri-MAN036	....		....		....
MK007331.1_COD_2018_Ituri-MAN002	....		....		....
MK007338.1_COD_2018_Ituri-MAN037	....		....		....
MK007334.1_COD_2018_Ituri-MAN020	....		....		....
MK007333.1_COD_2018_Ituri-MAN006	....		....		....
MK163644.1_COD_2018_Ituri-BEN017	....		....		....
MK163649.1_COD_2018_Ituri-BEN104	....		....		....
MK163650.1_COD_2018_Ituri-BEN114	....		....		....
MK163656.1_COD_2018_Ituri-BEN162	....		....		....
MK007330.1_COD_2018_Ituri-18FHV090	....		....		....
MK007341.1_COD_2018_Ituri-MAN042	....		....		....
MK731990.1_COD_2018_BEN164-MANA1-17	....		....		....
MK007342.1_COD_2018_Ituri-MAN046	....		....		....
MK007343.1_COD_2018_Ituri-MAN048	....		....		....
MK007332.1_COD_2018_Ituri-MAN003	....		....		....
MK007344.1_COD_2018_Ituri-MAN065	....		....		....
MK007340.1_COD_2018_Ituri-MAN040	....		....		....
MK007339.1_COD_2018_Ituri-MAN039	....		....		....
MK007335.1_COD_2018_Ituri-MAN025	....		....		....
MK088510.1_COD_2018_Ituri-MAN035	....		....		....
MK163645.1_COD_2018_Ituri-BEN018	....		....		....
MK163663.1_COD_2018_Ituri-BEN230	....		....		....
MK163646.1_COD_2018_Ituri-BEN020	....		....		....
MK163652.1 _COD_2018_Ituri-BEN141	....		....		....
MK731993.1_COD_2018_BEN167-MANA5-11	....		....		....
MK163653.1_COD_2018_Ituri-BEN143	....		....		....
MK163654.1_COD_2018_Ituri-BEN146	....		....		....
MK731992.1_COD_2018_BEN165-MANA3-20	....		....		....
MK163648.1_COD_2018_Ituri-BEN070	....		....		....
MK163651.1_COD_2018_Ituri-BEN140	....		....		....
MK007329.1_COD_2018_Ituri-18FHV089	....		....		....
MK163655.1_COD_2018_Ituri-BEN148	....		....		....
MK163664.1_COD_2018_Ituri-BEN234	....		....		....
MK163666.1_COD_2018_Ituri-BEN240	....		....		....
MK163671.1_COD_2018_Ituri-BEN269	....		....		....
MK163670.1_COD_2018_Ituri-BEN268	....		....		....
MK163673.1_COD_2018_Ituri-BEN286	....		....		....
MK163675.1_COD_2018_Ituri-BEN294	....		....		....
MK163674.1_COD_2018_Ituri-BEN292	....		....		....
MK163672.1_COD_2018_Ituri-BEN274	....		....		....
MK163665.1_COD_2018_Ituri-BEN236	....		....		....
MK163669.1_COD_2018_Ituri-BEN267	....		....		....
MK163667.1_COD_2018_Ituri-BEN255	....		....		....
MK163661.1_COD_2018_Ituri-BEN214	....		....		....
MK163668.1_COD_2018_Ituri-BEN263	....		....		....

Figure S2. Primers and probe described in the Gibb et al. assay matched against the multiple alignment dataset (glycoprotein gene) representing EBOV strains during the 2018 outbreak in the North Kivu and Ituri Provinces of the Democratic Republic of the Congo. Y = C/T; S = C/G; K = G/T. Nucleotide positions refers to the sequence of Zaire ebolavirus genome (GenBank accession number MK007339).
